# Supplementary figures and images for: Emergence of mcr-9.1 in Extended-Spectrum-β-Lactamase-Producing Clinical Enterobacteriaceae in Pretoria, South Africa: Global Evolutionary Phylogenomics, Resistome, and Mobilome
Source: mSystems. 2020 May 19;5(3):e00148-20. doi: 10.1128/mSystems.00148-20 (PMC7253365; doi:10.1128/mSystems.00148-20)

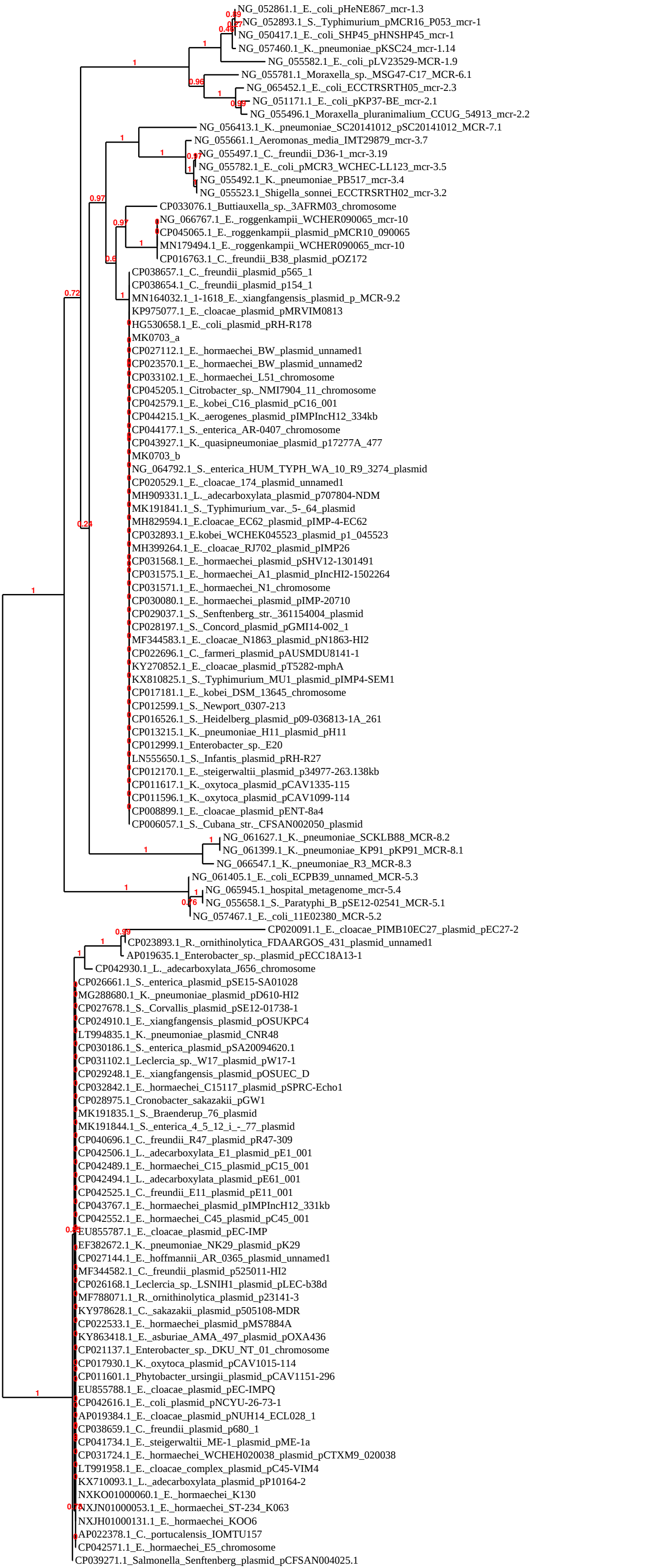

Supplement: FIG S1 [file mSystems.00148-20-sf001.pdf]
